# Management of Patients with Colorectal Cancer through Fast-Track Surgery

**DOI:** 10.3390/ijerph21091226

**Published:** 2024-09-18

**Authors:** Arianna Scala, Antonio D’Amore, Maria Pia Mannelli, Mario Mensorio, Giovanni Improta

**Affiliations:** 1 Department of Public Health, University Hospital of Naples Federico II, 80131 Naples, Italy; giovanni.improta@unina.it; 2 AORN “Antonio Cardarelli” Hospital, 80131 Naples, Italy; antonio.damore@aocardarelli.it (A.D.); mariapia.mannelli@aocardarelli.it (M.P.M.); mario.mensorio@aocardarelli.it (M.M.); 3 Interdepartmental Center for Research in Healthcare Management and Innovation in Healthcare (CIRMIS), University of Naples Federico II, 80131 Naples, Italy

**Keywords:** colorectal cancer, lean six sigma, lean thinking, value stream map, length of stay, health management

## Abstract

Colorectal cancer (CRC) is the third most common cancer in men and the second most common in women globally. CRC is considered a priority public health issue due to its incidence and the high associated costs. Surgery is the predominant therapeutic approach for CRC. Given the involvement of the intestinal tract in the surgical process, there is a significant increase in postoperative morbidity rates, and the average length of hospital stay (LOS) tends to lengthen. In this research, we employed the Lean Six Sigma (LSS) methodology, specifically utilizing the DMAIC cycle, to identify and subsequently examine the effects of fast-track surgery on hospitalization times for interventions related to CRC at the AORN “Antonio Cardarelli” Hospital in Naples (Italy). The process analysis, guided by the DMAIC cycle, facilitated a reduction in the median LOS from 14 days to 12 days. The most notable improvement was observed in the 66–75 age group without comorbidities. The LSS approach provides methodological rigor, as previously recognized, enabling substantial enhancements to the process. This involves standardizing outcomes, minimizing variability, and achieving an overall reduction in the LOS from 14 to 12 days.

## 1. Introduction

Colorectal cancer (CRC) ranks as the third most prevalent form of cancer among men, accounting for 746,000 cases or 10.0% of all cancers [1]. Among women, it holds the second position with 614,000 cases, representing 9.2% of total cancer diagnoses globally. Approximately 55% of these cases are reported in developed nations [1]. The incidence of colorectal cancer exhibits substantial geographical diversity, with remarkably similar patterns observed in both men and women. Worldwide, incidence rates display a tenfold variation in both genders, underscoring significant global disparities in this ailment. Due to the substantial human and financial toll associated with colorectal cancer, screening programs are strongly recommended to facilitate early detection and intervention when the disease is still in a treatable stage [2].

Numerous randomized controlled trials have substantiated the effectiveness of colorectal screening initiatives employing methods such as the fecal occult blood test (FOBT), sigmoidoscopy, or colonoscopy, demonstrating a significant reduction in both mortality and incidence. Specifically, four randomized controlled trials systematically assessed the impact of a screening program utilizing the Guaiac-FOBT on CRC-specific mortality [3,4,5,6]. The collective findings revealed a noteworthy 16% decrease in mortality within the screening group when compared to the non-screened control cohort [7]. Among these trials, only one reported a reduction in the cumulative incidence among the screened population [3], while the others observed either no discernible effect [4,5] or a marginal increase [6].

Surgery stands out as the predominant therapeutic approach for CRC. Given the intricate involvement of the intestinal tract in the surgical process, there is a notable elevation in postoperative morbidity rates, and the average duration of the postoperative length of hospital stay (LOS) tends to be extended [8]. In response to these challenges, numerous innovative techniques and methods have been introduced in the realm of colorectal cancer surgery [9]. The advent of laparoscopic (assisted) surgery for colorectal resection marked a significant milestone, with its initial description by Jacobs et al. in 1991 [10]. Laparoscopic colorectal cancer surgery offers distinct advantages, notably in the reduction in the average postoperative LOS and postoperative morbidity [11,12].

In contemporary health systems, considerable emphasis is placed on sustainability, with a focus on managing costs in relation to economic resources while maintaining high service quality. In this context, “fast-track surgery” (FTS), also known as “Enhanced Recovery After Surgery” (ERAS), has gained prominence as a strategy to accelerate patient recovery, reduce postoperative morbidity, and shorten the length of hospital stay [13,14].

FTS signifies an expedited route within the realm of surgery, involving the acceleration of timelines across all stages of hospitalization, from the preoperative phase to the postoperative phase. In essence, FTS is a multidisciplinary approach grounded in the concept that the perioperative phase encompasses various medical specializations, including anesthesia, nutrition, and rehabilitation. The overarching goal of FTS is to diminish operative stress, alleviate patient discomfort, and hasten the recovery process [15]. Effective FTS implementation requires a well-defined protocol that includes measures such as pain management and the control of bleeding, which have been shown to significantly improve patient outcomes and reduce healthcare costs [16,17,18].

In order to develop an effective FTS program, a rigorous methodology must be used. Numerous process improvement strategies, spanning from simulation models [19,20] to principles of quality management [21,22], have been explored. Notably, Lean Six Sigma (LSS) has emerged as a successful approach within the healthcare context. LSS blends the analytical strength inherent in Six Sigma with lean thinking principles, particularly the emphasis on achieving “zero waste” [23]. Initially applied primarily in the manufacturing industry, LSS has gained substantial traction as a managerial methodology in the healthcare sector, with a diverse range of applications over the past two decades [24,25]. Van Lent et al. implemented a lean thinking approach in a hospital-based chemotherapy day unit, resulting in an enhanced process design and increased efficiency, leading to a more timely delivery of care [26]. In a separate study, Bisgaard et al. focused on reducing the length of stay for patients with chronic obstructive pulmonary disease. Their findings demonstrated the potential to enhance the quality of care while concurrently reducing costs [27]. Fiorillo et al., employing the principles of lean thinking, targeted the identification and elimination of waste and inefficiencies in preoperative activities, with a particular focus on the preoperative LOS as a key performance indicator. Utilizing the Ishikawa diagram, the main causes of these inefficiencies were pinpointed, facilitating a thoughtful consideration of potential solutions. A primary corrective measure involved the implementation of a pre-hospitalization service. A comparative statistical analysis underscored the significance of the implemented solutions [28]. Mahesh et al. utilized the DMAIC cycle of LSS to reduce patient wait times in a cardiology outpatient department, and other studies successfully applied LSS to optimize care pathways for surgical and medical patients, achieving improvements in care quality and cost reduction [29,30,31,32].

The aim of this study is to implement and evaluate the introduction of an FTS protocol for patients undergoing surgery for CRC by applying the DMAIC cycle. This approach will leverage the strengths of LSS to systematically enhance postoperative recovery, reduce morbidity, and shorten the LOS, contributing a novel perspective to the optimization of CRC surgical care.

## 2. Materials and Methods

This project was conducted at the Complex Operative Unit of General Surgery at the AORN “Antonio Cardarelli” Hospital in Naples. Data for all patients included in this study were gathered from the hospital’s digital information system database, encompassing anamnestic details (such as age and gender) and clinical variables (admission, surgery, and discharge dates, as well as comorbidities). Statistical analyses were performed using Matlab 2022b.

### 2.1. Define

A multidisciplinary team undertook this investigation within the Complex Operative Unit of General Surgery at the AORN “Antonio Cardarelli” Hospital. The initial phase involved the development of a project charter (Table 1) to establish a collective understanding of this project’s specifics, including critical-to-quality (CTQ) aspects, research questions, objectives, items within and outside the project scope, and the timeline.

Furthermore, a SIPOC (Supplier, Input, Process, Output, Customer) diagram was constructed to elucidate the key characteristics of the main process and define this project’s scope (Table 2).

### 2.2. Measure

In the define phase, the interdisciplinary team pinpointed the key distinctive features of this work, including the problem to be addressed, CTQ factors, and the methods to be employed. In the measure phase, measurements were conducted to assess the performance of the current process.

During this phase, the dataset was derived from a sample consisting of 255 patients who underwent surgery for colorectal cancer between January 2017 and December 2019. Following the implementation of the new protocol from January 2020 to January 2022, information was gathered from a sample of 134 patients to assess the impact of improvement measures on the LOS.

The following information was collected for each patient:

- Age;
- Gender;
- Type of admission;
- Ward of admission;
- Type of tumor;
- Diabetes;
- Hypertension;
- Abdominal Adherences;
- Cardiological disorders;
- Respiratory disorders;
- Gallstones;
- Peritonitis;
- Other tumors;
- Complicated procedure;
- Mode of discharge.

The aim of this project was to reduce the hospital stay for patients who underwent a high-priority elective intervention (priority “A”). Interventions classified as ‘urgent’ were excluded. The inclusion criteria were as follows (with ICD-9-CM ver. 2007 code):

- Surgical procedures:
  ▪ 4571 multiple segmental resection of the large intestine;
  ▪ 4572 cecum resection;
  ▪ 4573 right hemicolectomy;
  ▪ 4574 transverse colon resection;
  ▪ 4575 left hemicolectomy;
  ▪ 4576 sigmoidectomy;
  ▪ 4579 other partial resection of the large intestine;
  ▪ 458 total intra-abdominal colectomy;
  ▪ 485 rectum resection by the abdominoperineal route;
  ▪ 4861 transaxillary rectosigmoidectomy;
  ▪ 4862 anterior resection of the rectum with concomitant colostomy;
  ▪ 4863 other anterior resection of the rectum;
  ▪ 4864 posterior resection of the rectum;
  ▪ 4865 resection of the rectum according to Duhamel;
  ▪ 4869 other resection of the rectum.
- A main or secondary diagnosis of the following:
  ▪ 1530 malignant tumors of the hepatic flexure;
  ▪ 1531 malignant tumors of the transverse colon;
  ▪ 1532 malignant tumors of the descending colon;
  ▪ 1533 malignant tumors of the sigma;
  ▪ 1534 malignant tumors of the cecum;
  ▪ 1535 malignant tumors of the appendix;
  ▪ 1536 malignant tumors of the ascending colon;
  ▪ 1537 malignant tumors of the splenic flexure;
  ▪ 1538 malignant tumors of other (specified) sites of the large intestine;
  ▪ 1540 malignant tumors of the rectosigmoid junction;
  ▪ 1541 malignant tumors of the rectum;
  ▪ 1542 malignant tumors of the anal canal;
  ▪ 1543 malignant tumors of the anus, unspecified;
  ▪ 1548 other malignant tumors of the rectum, rectosigmoid junction, and anus.

Both graphs and descriptive variables were used to assess the current status of the CTQ in various study variables. For the graphical part, Microsoft Excel (MS Office 2016 suite) (Microsoft Corp, Redmond, WA, USA) was used from the sheet where the study variables and patient data were collected. In this paper, Run Chart was used to observe the preoperative LOS, postoperative LOS, and recorded LOS for the various samples. A script developed in the Matlab environment (R2022b) was instead used to calculate descriptive statistics (mean, median, minimum, maximum, and standard deviation) for the LOS in each of the subgroups identified from the different values that the variables listed above could assume.

**Figure 1:**
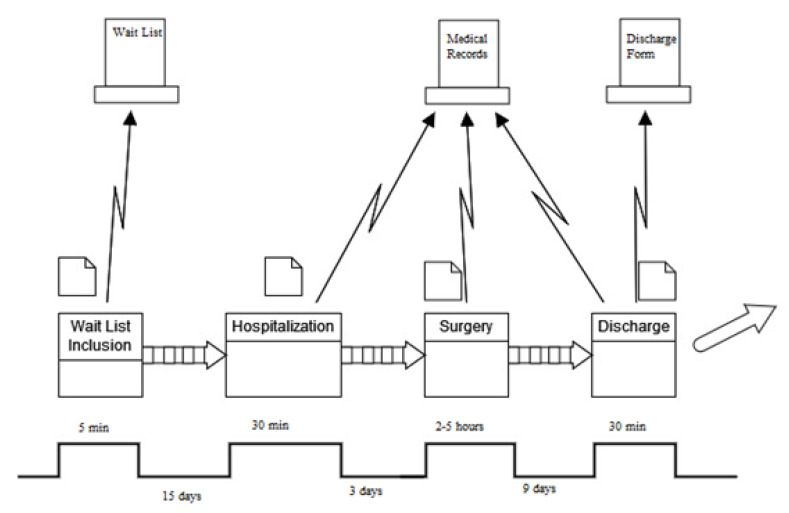
Value stream map (AS-IS).

Furthermore, the progression of the process was additionally charted utilizing a value stream map (VSM) to observe and comprehend the movement of materials and information, gaining insight into the existing state of the process. VSMs enable the visualization of the considered process by pinpointing tasks, execution durations, and personnel involvement (Figure 1).

### 2.3. Analyze

In the analyze phase, starting from the measurements made in the previous section and the critical evaluation of the VSM, it is possible to discuss the critical issues highlighted within the process. To conduct this, the multidisciplinary team involved in this project used a structured analysis approach such as the use of the Ishikawa diagram. This diagram, also called a fishbone diagram, presents on the head the main problem to be addressed, in this case an increase in the LOS, while on the fishbones the main causes and all the sub-causes that contributed to the CTQ. The de-composition of the problem was conducted by combining the diagram with the use of the ‘5 Why’ technique. Only through this approach is it possible to identify effective corrective actions, the subject of the next step.

### 2.4. Improve

In this phase, a new FTS protocol was defined, covering the entire process from pre-hospitalization to discharge. The first phase involves the patient and their relatives/companions meeting with the multidisciplinary team, including the surgeon, anesthetist, and nurses, to receive information about pre-admission, education, and counseling. Indeed, it the positive impact that detailed, procedure-specific, patient-centered information sessions can have on the entire surgical experience was demonstrated [33,34]. The second phase concerns “preoperative nutritional care”. Malnutrition before surgery has been linked to higher rates of postoperative complications and mortality, along with unfavorable outcomes in the treatment of gastrointestinal cancer [35]. Conducting a preoperative nutritional assessment to identify apparent or subtle malnutrition provides a chance to enhance nutritional status and address specific deficiencies [36]. With the preoperative phase completed, in the perioperative phase, attention is directed towards anesthesia, antiemetics, fluid management, and infection control. Subsequently, postoperatively, crucial areas of focus include early mobilization, analgesia, antiemetics, fluid management, nutrition, and urinary catheterization [37].

### 2.5. Control

In the last phase, the same variables were collected in the period following the actions in the improve phase. This stage includes data from 134 patients who were discharged after a priority “A” procedure for colorectal cancer from January 2020 to January 2022.

Graphical tools and statistical analysis were used to compare how the CTQ changed in the two groups. 

For graphical tools, Microsoft Excel was always used, implementing Run Chart on the post-intervention data.

The recognition of the FTS protocol enabled a decrease in the duration of activities that do not contribute significant value. More precisely, following the completion of this project, the schedule was reiterated, resulting in the development of a new VSM.

For the statistical analysis, on the other hand, a special script was created in the Matlab environment. After reading the dataset from a Microsoft Excel file, the code used a specially created Boolean variable from the year of discharge to create the two groups to be compared. At this point, subgroups were created for each variable included in this study according to the possible alternatives available, i.e., male and female for the variable gender, which have the LOS as the observation variable. In these subgroups, the One-sample Kolmogorov–Smirnov test was first implemented to assess the normality of the distribution. The subsequent processing was based on the execution of a conditional if construct. That is, if normality was verified, the statistical *t*-test was implemented; otherwise, a two-sided Wilcoxon rank sum test was employed. For both, a 95% confidence interval was chosen. The difference was considered statistically significant when the *p*-value was less than 0.05.

Finally, the same code was used for descriptive statistics.

## 3. Results

According to the steps of the DMAIC cycle, after defining the project, timeframe, and stakeholders and actors involved, the as-is of the process was evaluated. The first data that were analyzed were the descriptive statistics. It should be noted that for the purposes of the analysis, as the distributions are not normal, we would reason in terms of the median. The results are shown in Table 3.

The median in the dataset is 14 days for almost all variables. Higher values were recorded for patients whose main diagnosis was tumors of the large abdominal organs (such as liver and pancreas), with respiratory disorders among the comorbidities, or who were discharged with ‘Transferred to another regimen’ or ‘Voluntary’. The variability in these cases, obtained by looking at the standard deviation, was, however, among the highest. 

**Figure 2:**
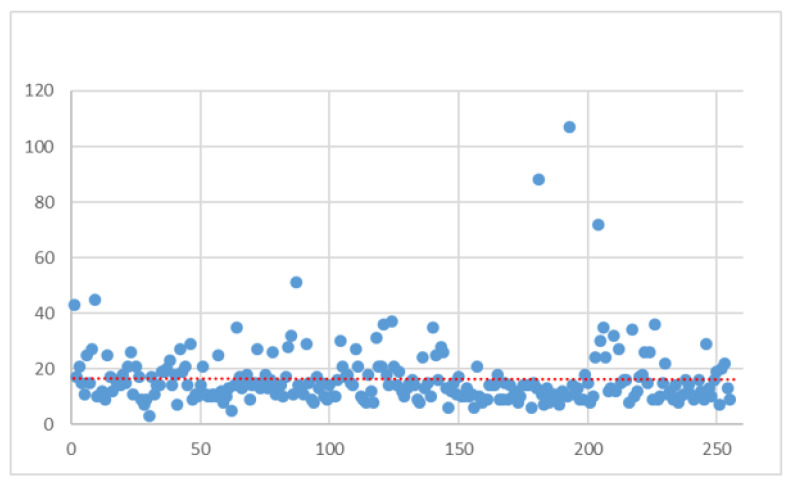
LOS distribution (AS-IS).

From a graphical point of view, Figure 2 shows the distribution of the LOS within the group under investigation.

It can be seen from the figure that there are several outliers that deviate from the standard distribution tendency. The red line in the figure represents the linear trend line of the observed data. It can be seen that a trend towards a reduction in the length of stay was observed in the last patients treated and analyzed. 

**Figure 3:**
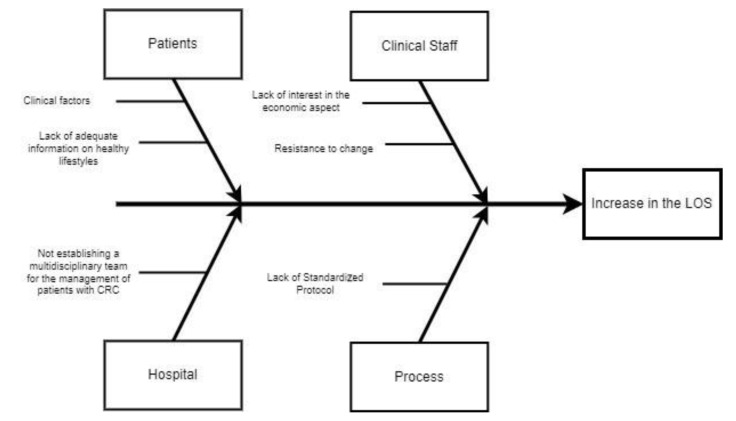
Ishikawa diagram.

After this measurement phase, the causes of this variability were investigated through brainstorming sessions with all staff involved. Four primary causes were found: patients, clinical staff, hospital, and the process. Ishikawa’s analysis (Figure 3), through the “problem–analysis–solution” pathway, enabled us to realize that each root cause had secondary causes. The most relevant were identified as the following: the lack of adequate information on healthy lifestyles, absence of an officially defined multidisciplinary team, and lack of a standardized protocol.

Once the corrective actions were identified and implemented, the variables of interest were collected for the newly admitted patients according to the inclusion criteria defined in the measure phase over the next two years to test the effectiveness of the solutions implemented. As before, the first results analyzed were the descriptive statistics. Table 4 shows the results obtained.

**Figure 4:**
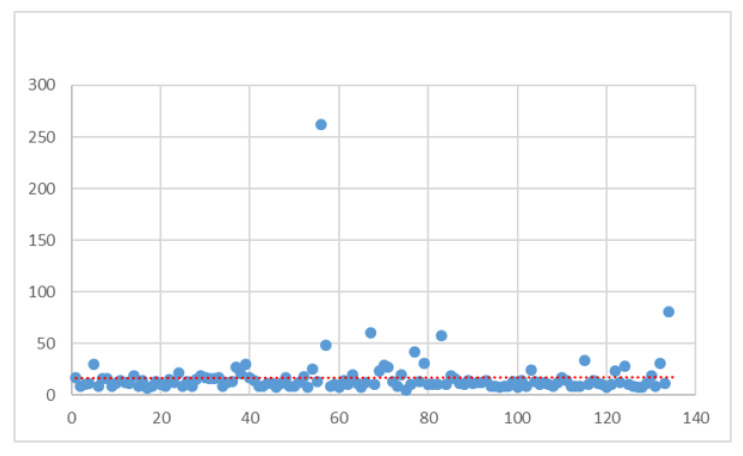
LOS distribution (TO BE).

As can be seen from the results in the table, a reduction in the median was observed in the total sample from 14 to 12 days. An interesting fact is that the age distribution shows an increase in the LOS for younger patients. Figure 4 shows the LOS trend in the group under investigation.

The benefits of corrective actions are immediately evident from the distribution. In addition to the linear prediction line tending to decrease, there were fewer outliers, with values more concentrated towards a lower LOS. These results were then confirmed by statistical analysis. As anticipated, no normal trend was obtained for any of the identified subgroups, so the two-sided Wilcoxon rank sum test was always implemented. The results are shown in Table 5.

Statistical analysis showed a statistically significant reduction in the LOS over the entire sample. In addition, significant reductions were obtained for patients with 66 ≤ age ≤ 75; males; patients admitted to surgical wards; patients discharged home and not presenting comorbidities such as Diabetes, Hypertension, Abdominal Adherences, respiratory disorders, Gallstones, Peritonitis, or other tumors; and discharged with complicated DRG. Another significant finding is that voluntary discharges were reduced to zero and that the enhancement in pre-hospitalization had real benefits on hospitalization times.

**Figure 5:**
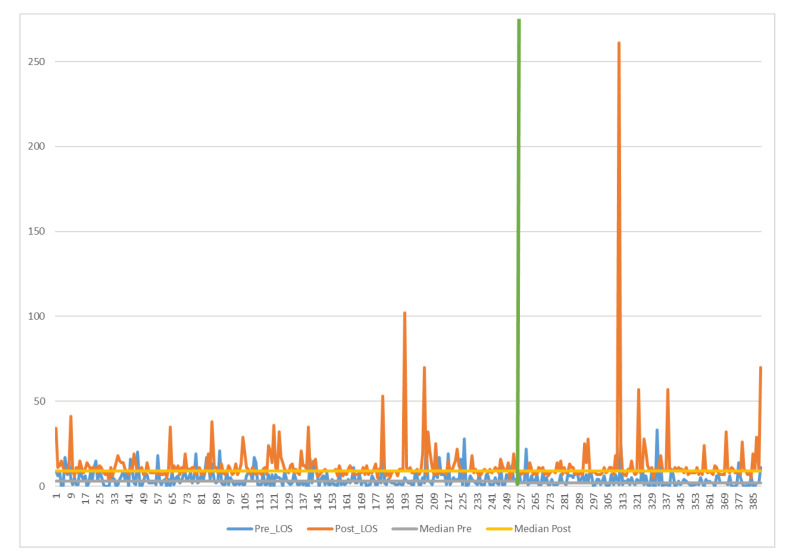
Pre-Op and Post-Op LOS distribution. Green line: separates pre-improvement sample from post improvement sample.

To understand better this phenomenon, it was decided to also graphically represent the trend in the Pre-Op LOS and Post-Op LOS. The results are shown in Figure 5.

**Figure 6:**
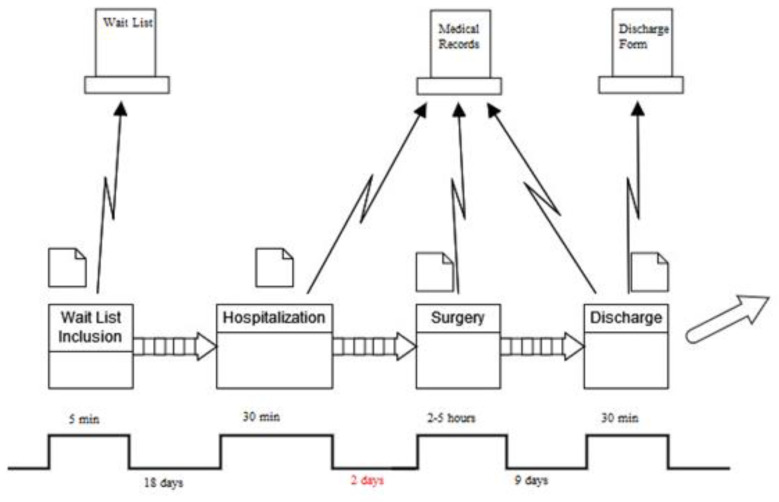
Value stream map (TO BE).

In Figure 6, indeed, it is possible to observe the new VSM illustrating how detailed information sessions, tailored to the procedure and centered around the patient, contributed to enhancing pre-hospitalization activities. This transition resulted in a median value decrease from 3 days to 2 days post-improvement.

**Figure 7:**
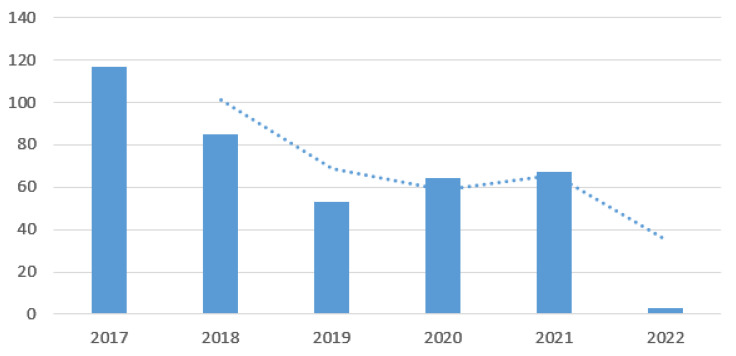
Patients treated per year.

Figure 7 also shows another key aspect. Thanks to the reorganization of the process, it became more attractive to patients who chose to be treated in the hospital.

The histogram shows that from 2017, there was a rapid reduction in the number of patients treated. From 2019, this trend reversed, showing an increase in the number of patients admitted, as can also be seen from the moving average trend line. The decrease in 2022 is due to the small number of treated cases included in this study, having stopped observations in January 2022.

## 4. Discussion

This study demonstrates the initial impact of applying the DMAIC cycle of Lean Six Sigma to improve the management of colorectal cancer patients undergoing fast-track surgery. At baseline, the median length of stay (LOS) was 14 days, higher than the 12-day average reported in the literature for traditional surgery [38]. Evenafter implementing fast-track surgery, the median LOS was only reduced to 12 days, which remains significantly above the 6-day average observed in other hospitals with similar protocols [39].

The data revealed that a higher LOS was particularly pronounced in patients with complex conditions, such as tumors of large abdominal organs or comorbidities like respiratory disorders. Improvements were seen, such as a reduction in the number of outliers and a more concentrated LOS distribution, particularly among patients without major comorbidities and those discharged home. However, ongoing organizational challenges, especially in the preoperative phase, and the lack of sufficient staff to manage the entire protocol have limited our ability to achieve more substantial reductions.

This study marks only the first phase of implementing the DMAIC methodology. Future developments will focus on addressing these barriers to further reduce the LOS and bring it closer to the averages reported in the literature. As the LOS is reduced, we also plan to collect information on patient satisfaction and long-term outcomes to better evaluate the overall effectiveness of the implemented changes.

## 5. Conclusions

In this document, the LSS methodology was employed to diminish the length of stay (LOS) for patients undergoing colorectal surgery at the AORN “Antonio Cardarelli” Hospital in Naples. The DMAIC cycle was utilized to achieve this objective. In the define phase, the key features of this project were specified and illustrated through a project charter and a SIPOC diagram. During the measure phase, the existing process was assessed based on the median and standard deviation. A VSM of the current workflow was drafted to identify activities and their corresponding median times. The VSM facilitated the identification of value-added activities and wastages. In the analysis phase, the reasons for a prolonged LOS were explored, and statistical analyses were conducted to pinpoint the variables exerting the most influence on the LOS. Subsequently, the implemented FTS was detailed in the improve phase. Finally, the control phase demonstrated that the attained results were sustained. The noteworthy outcomes obtained reveal a reduction in the median LOS from 14 to 12 days. In comparison to the relevant literature in the field and analogous clinical pathways, the present study yielded compelling and valuable results, offering a valuable guide for healthcare quality improvement endeavors. Nevertheless, it is crucial to emphasize, as indicated in the literature, that the intrinsic characteristics and conditions of the organization in which the clinical pathway is implemented should be carefully considered, as they may significantly impact the outputs and outcomes of the process. 

## 6. Authors’ Opinion

This study is significant because it demonstrates how an approach originally developed for the manufacturing industry, Lean Six Sigma, can be effectively applied to healthcare processes as well. Lean Six Sigma is known for its structured, data-driven methodology, which focuses on reducing waste, minimizing variation, and enhancing overall efficiency. These principles, while traditionally associated with manufacturing, have proven to be just as relevant and impactful in healthcare settings, where process improvements can directly translate into better patient outcomes, reduced costs, and a higher quality of care.

What makes Lean Six Sigma particularly different from other process improvement approaches is its unique combination of two powerful strategies: “Lean”, which aims to eliminate non-value-added activities, and “Six Sigma”, which focuses on reducing variability and improving quality through statistical methods. This dual approach sets Lean Six Sigma apart by providing a comprehensive framework that not only targets inefficiencies but also ensures that processes are consistent, reliable, and sustainable over time.

By applying this rigorous methodology to healthcare, the study illustrates how Lean Six Sigma can adapt to various sectors beyond its original industrial context, making it a flexible tool for driving change in complex environments. Unlike other approaches that may focus solely on qualitative measures or incremental changes, Lean Six Sigma provides a systematic and quantitative foundation for identifying problems, measuring impacts, and implementing lasting improvements. This adaptability and focus on measurable results demonstrate why Lean Six Sigma is a valuable strategy for improving healthcare processes and suggest that its use could be expanded to many other areas where efficiency and quality are paramount.

## Figures and Tables

**Table 1:** Project charter.

Project Title Fast-Track Surgery for Colorectal Cancer	
**Problem statement** An inappropriate prolongation of the length of hospital stay for patients undergoing surgery for colorectal cancer	**Objective statement** Introduce a clinical pathway that can solve the presented problem
**Critical to quality** The CTQ is the duration of the length of hospital stay	**Target** Realize a corrective measure to reduce the CTQ
Project leader: Names will be added if the paper is accepted Project champion: Team members:	
**Timeline** Define → May–August 2018 Measure → September–October 2018 Analyze → November 2018–April 2019 Improve → May–June 2019 Control → January 2020–December 2022	
**In scope** Surgery for colorectal cancer Complex Operative Unit of General Surgery	**Out of scope** Any other type of intervention All other structures

**Table 2:** SIPOC diagram.

Supplier	Input	Process	Output	Customer
Complex Operative Unit of General Surgery	Surgical and medical services.	Care process: Pre-hospitalization; Surgery; Postoperative activities; Discharge.	Improved outcome for patients. Diagnostic and therapeutic information. Health.	Patients AORN “Antonio Cardarelli” Hospital

**Table 3:** Descriptive statistics (AS-IS).

Variable	Value	N	Standard Deviation	Min	Max	Median
All	All	255	11.01	3	107	14
Age	Under 50	18	8.13	6	32	13
	50 ≤ Age ≤ 65	76	10.85	7	88	12
	66 ≤ Age ≤ 75	90	10.19	6	72	14
	Over 75	71	12.75	3	107	14
Gender	Male	140	11.20	3	88	14
	Female	115	10.79	5	107	14
Type of Admission	Scheduled	63	13.47	8	88	14
	Scheduled with pre-hospitalization	192	10.02	3	107	14
Ward of Admission	Other surgeries	234	10.70	3	107	14
	Specialized ward	21	14.31	7	72	13
Type of Tumor	Colon	107	8.97	3	72	14
	Rectum–sigma	130	12.71	6	107	14
	Abdominal organs	4	7.05	10	27	20.5
	Other abdominal tumors	14	8.81	5	36	10.5
Diabetes	No	243	11.21	3	107	14
	Yes	12	6.05	8	30	15
Hypertension	No	229	11.48	3	107	14
	Yes	26	5.18	7	27	14
Abdominal Adherences	No	244	9.45	3	88	14
	Yes	11	29.06	8	107	11
Cardiological Disorders	No	204	9.76	5	88	13
	Yes	51	14.61	3	107	16
Respiratory Disorders	No	227	9.53	5	88	13
	Yes	28	18.12	3	107	21
Gallstones	No	248	11.12	3	107	14
	Yes	7	6.32	9	27	13
Peritonitis	No	234	8.21	3	88	14
	Yes	21	23.82	8	107	24
Other Tumors	No	234	11.37	3	107	14
	Yes	21	5.63	7	27	14
Complicated Procedure	No	138	11.99	5	107	12
	Yes	117	9.38	3	72	16
Mode of Discharge	Dead	9	12.98	3	45	24
	To home	239	7.97	5	72	14
	Voluntary	5	33.24	6	88	19
	Transferred to another institution	0	0.00	0	0	0
	Transferred to another regime	2	64.35	16	107	61.5
	Transferred to rehabilitation institution	0	0.00	0	0	0

**Table 4:** Descriptive statistics (TO-BE).

Variable	Value	N	Standard Deviation	Min	Max	Median
All	All	134	23.80	5	262	12
Age	Under 50	7	9.69	9	34	17
	50 ≤ Age ≤ 65	42	9.95	8	58	11.5
	66 ≤ Age ≤ 75	52	11.61	5	81	11
	Over 75	33	44.10	8	262	13
Gender	Male	87	28.77	5	262	11
	Female	47	9.39	8	58	13
Type of Admission	Scheduled	24	12.42	8	58	16
	Scheduled with pre-hospitalization	110	25.64	5	262	11
Ward of Admission	Other surgeries	124	10.84	5	81	11
	Specialized ward	10	77.86	9	262	15.5
Type of Tumor	Colon	64	31.68	7	262	12
	Rectum–sigma	60	13.84	8	81	11
	Abdominal organs	1	0.00	25	25	25
	Other abdominal tumors	9	8.48	5	34	10
Diabetes	No	128	24.32	5	262	12
	Yes	6	1.60	9	13	11
Hypertension	No	123	24.73	5	262	12
	Yes	11	8.20	8	31	10
Abdominal Adherences	No	124	24.60	5	262	11.5
	Yes	10	10.05	9	42	12.5
Cardiological Disorders	No	116	25.31	5	262	12
	Yes	18	9.94	9	42	11.5
Respiratory Disorders	No	126	8.49	5	60	12
	Yes	8	86.35	9	262	21
Gallstones	No	129	24.23	5	262	11
	Yes	5	7.05	9	28	16
Peritonitis	No	123	24.69	5	262	12
	Yes	11	9.71	9	42	12
Other Tumors	No	129	24.24	5	262	11
	Yes	5	5.52	11	25	16
Complicated Procedure	No	91	27.69	7	262	11
	Yes	43	12.29	5	60	12
Mode of Discharge	Dead	2	0.00	10	10	10
	To home	129	8.91	5	60	12
	Voluntary	0	0.00	0	0	0
	Transferred to another institution	1	0.00	31	31	31
	Transferred to another regime	0	0.00	0	0	0
	Transferred to rehabilitation institution	2	127.99	81	262	171.5

**Table 5:** Results of statistical comparison.

Variable	Value	Pre-Improvement		Post-Improvement		*p*-Value
		N	Median	N	Median	
All	All	255	14	134	12	**0.014**
Age	Under 50	18	13	7	17	0.627
	50 ≤ Age ≤ 65	76	12	42	11.5	0.273
	66 ≤ Age ≤ 75	90	14	52	11	**0.012**
	Over 75	71	14	33	13	0.453
Gender	Male	140	14	87	11	**0.003**
	Female	115	14	47	13	0.907
Type of Admission	Scheduled	63	14	24	16	0.693
	Scheduled with pre-hospitalization	192	14	110	11	**0.007**
Ward of Admission	Other surgeries	234	14	124	11	**0.004**
	Specialized ward	21	13	10	15.5	0.385
Type of Tumor	Colon	107	14	64	12	0.211
	Rectum–sigma	130	14	60	11	0.050
	Abdominal organs	4	20.5	1	25	0.800
	Other abdominal tumors	14	10.5	9	10	0.611
Diabetes	No	243	14	128	12	**0.035**
	Yes	12	15	6	11	**0.020**
Hypertension	No	229	14	123	12	**0.021**
	Yes	26	14	11	10	0.443
Abdominal Adherences	No	244	14	124	11.5	**0.010**
	Yes	11	11	10	12.5	0.750
Cardiological Disorders	No	204	13	116	12	0.102
	Yes	51	16	18	11.5	0.077
Respiratory Disorders	No	227	13	126	12	**0.044**
	Yes	28	21	8	21	0.909
Gallstones	No	248	14	129	11	**0.007**
	Yes	7	13	5	16	0.318
Peritonitis	No	234	14	123	12	**0.042**
	Yes	21	24	11	12	0.099
Other Tumors	No	234	14	129	11	**0.009**
	Yes	21	14	5	16	0.696
Complicated Procedure	No	138	12	91	11	0.524
	Yes	117	16	43	12	**0.016**
Mode of Discharge	Dead	9	24	2	10	0.145
	To home	239	14	129	12	0.016
	Voluntary	5	19	0	0	-
	Transferred to another institution	0	0	1	31	-
	Transferred to another regime	2	61.5	0	0	-
	Transferred to rehabilitation institution	0	0	2	171.5	-

## Data Availability

The data presented in this study are available from the authors upon reasonable request.
